# Improving production and quality of life for smallholder farmers through a climate resilience program: An experience in the Brazilian *Sertão*

**DOI:** 10.1371/journal.pone.0251531

**Published:** 2021-05-21

**Authors:** Alexandre Gori Maia, Jennifer Anne Burney, José Daniel Morales Martínez, Daniele Cesano

**Affiliations:** 1 Center for Applied Economics, Agricultural and Environmental Research, University of Campinas, Rua Pitagoras, Brazil; 2 School of Global Policy and Strategy, University of California, San Diego, La Jolla, CA, United States of America; 3 Adapta Group, Rio de Janeiro, Brazil; Szechenyi Istvan University, HUNGARY

## Abstract

We use a combination of economic and wellbeing metrics to evaluate the impacts of a climate resilience program designed for family farmers in the semiarid region of Brazil. Most family farmers in the region are on the verge of income and food insufficiency, both of which are exacerbated in prolonged periods of droughts. The program assisted farmers in their milk and sheepmeat production, implementing a set of climate-smart production practices and locally-adapted technologies. We find that the program under evaluation had substantive and significant impacts on production practices, land management, and quality of life in general, using several different quasi-experimental strategies to estimate the average treatment effect on the treated farmers. We highlight the strengths and limitations of each evaluation strategy and how the set of analyses and outcome indicators complement each other. The evaluation provides valuable insights into the economic and environmental sustainability of family farming in semiarid regions, which are under growing pressure from climate change and environmental degradation worldwide.

## 1. Introduction

Family farming, both in Brazil and globally, is under tremendous pressures from climate change and environmental degradation, both of which are often in a positive (detrimental) feedback cycle [1]. In semiarid regions, where social and climate vulnerability tends to be widespread, minor changes in the environment may have harmful impacts on water supply and local food security [2, 3]. Economic pressures (most family farmers are poor) also mean that transient shocks can lead to long-run negative impacts, where farmers must deplete natural capital stock to cope with decreasing productivity.

Studies have highlighted how adaptive and mitigative strategies may simultaneously bring climate resilience and improve farmers’ quality of life. Farmers may adapt as a response to climate change by adjusting planting and harvesting dates, changing crop species, or improving agricultural practices and infrastructure [4]. The adoption of regenerative farming practices has also shown positive impacts on the food and nutrition security of impoverished farmers [5]. Basic strategies, such as the adoption of machinery and the access to technical training, have shown the ability to attenuate the negative impacts of climate change on agricultural production of family farms in the Brazilian semiarid [6]. Agroecological strategies that enhance the ecological resiliency of farming systems may also mitigate potential impacts of climate change, for example, through reduced soil erosion and degradation, provision of litter for organic material and soil nutrients, and reduced greenhouse gas emissions due to lower use of pesticides and fertilizers [7]. Farmers may benefit from reforestation and agroforestry systems through, for example, the trade of carbon credits, improved soil conservation, and the sustainable management of natural vegetation [8]. In general, although climate-smart agricultural systems targeted to impoverished farmers have been implemented worldwide by multilateral organizations, studies that empirically evaluate the effectiveness of such programs remain scarce.

Here we evaluate the impacts of a climate resilience program on the production practices and quality of life of smallholder family farmers in the Brazilian semiarid region (*Sertão*). The program under evaluation (the *Módulo Agroclimático Inteligente e Sustentátvel*, hereafter MAIS) was implemented on 100 farms in the Sertão between 2016 and 2018, aiming to improve food security, agricultural productivity, and attain environmental sustainability among family farmers in the *Jacuípe* Basin, Bahia state in Brazil [9, 10]. The region presents one of the highest levels of poverty and food insecurity in the country, and its main economic activity, dairy farming, has already been adversely affected by increasing temperatures and recurrent droughts [11].

The MAIS is a set of agricultural production practices and technologies with specific goals to improve milk and sheep meat yields. The program helps to support smallholder livestock and dairy farmers through both seasonal and longer-run climate variability by teaching farmers to grow extra forage and manage herds appropriately, while also regenerating and protecting their natural capital assets. The practices introduced by the MAIS program also include achieving and maintaining compliance with the Brazilian Forest Code, which states that 20% of native habitat in semiarid regions must be maintained and conserved.

We analyze the impacts of the MAIS program using data we gathered on labor and technology use, production practices, land management, farm income, and subjective wellbeing (SWB) among family farmers in the Sertão. Our analyses are based on two samples: a cross-sectional survey applied among 95 MAIS and 107 non-MAIS farmers after the intervention, and a panel dataset containing a sub-sample of 26 MAIS and 87 non-MAIS farmers interviewed before and after the intervention. We control for the lack of randomness in the designation of beneficiary farmers using the main identification strategies available for quasi-experimental designs: traditional difference-in-difference (DID) estimators; two-stage (2S) regression estimators; and methods derived from the propensity score (PS). We also decompose the total difference between the MAIS and non-MAIS farmers into (i) differences due to observable characteristics, such as the access to technology and better production practices implemented by the MAIS program; and (ii) differences due to unobservable differences between MAIS and non-MAIS farmers to better understand program impacts and selection effects.

This study provides an important case to understand the socioeconomic impacts of a program that has the potential to be expanded to family farmers in other semiarid regions. To our knowledge, this is the first study to evaluate the effectiveness of a climate resilience program designed for dairy and sheep meat farming. Moreover, the non-experimental design of most social programs in the developing world creates similar series of empirical challenges for evaluation to the ones we faced; we provide a roadmap for leveraging existing programs to learn more about which programs work and which do not, even when experimental implementation falls apart.

## 2. Background

### 2.1. Building sustainable and resilient agriculture in *Sertão*

The Brazilian *Sertão* provides a unique opportunity to analyze how specific climate resilience strategies may improve the quality of life for small-scale farmers. It is the most populous semiarid region of the world, home to roughly 20 million people in 2010 [12]. The main biome of the *Sertão* is known as *caatinga*, which extends over 900,000 km^2^ (10% of the country) and presents patterns of anomalous precipitation with occasional multi-year droughts [13]. The biome presents a rich biodiversity, with high levels of endemism [14]. However, poor land use practices, growing rates of deforestation, and prolonged periods of drought have compromised human development in the region.

A long-term sustainable development in the *Sertão* means finding a balance between ecosystem conservation and agricultural production. The region is home to 1.8 million farmers in Brazil (36% of the total 5.1 million), and two-thirds of them had a total value of production lower than 5,000 Brazilian reais (1,500 dollars) in 2016 (only 26% in Brazil) [15]. Most family farmers in the region are on the verge of income and food insufficiency, which tend to be exacerbated by climate change. In the *Jacuípe* Basin–a subset of the region in Bahia State–the average temperature increased by more than 2°C over the past 40 years, while the average precipitation fell between 300 and 450 mm, which corresponds to a reduction of 30% [11]. The main economic activity in the region is livestock and dairy farming, which is directly exposed to climate conditions in several ways. First, the animals themselves are exposed to droughts and heat stresses, and dairy production, in particular, is sensitive to temperature changes [6]; second, environmental conditions determine the amount of forage produced, which affects animal health and growth. In the long run, the progressive replacement of the native *caatinga* vegetation with grass pasture over time has also reduced farmers’ resilience to climate changes by decreasing the water retention capacity of the soil and its microbial biomass, and by further exposing animals by removing tree cover [16, 17].

Climate change also means that farmers must adapt to new environmental conditions through climate-resilient agriculture, using strategies that are able to recover from climate impacts in an effective manner. Resilient-agriculture in the Sertão requires, above all, farming systems that optimize the use of the water generated by low and unpredictable rainfall, increases the water storage in the soil, and the use of drought-tolerant crops [18]. For example, the integration between agriculture, livestock, and forest in Sertão has shown to present soils that are more resistant and resilient to prolonged droughts, especially the surface soil [19]. The extensive livestock production prevailing in the *Sertão* presents the lowest stocking rates in Brazil, and are primarily dependent on local natural resources [20]. Therefore, the preservation of the natural vegetation might be fundamental to sustain the long-term cattle raising in the region.

The MAIS program is an example of climate-resilient strategies for agriculture in the *Sertão*. The MAIS is a set of climate-smart production practices and locally-adapted technologies designed as a whole to be both resilient to climate variations and regenerative of the natural ecosystem [9]. In terms of land management, the MAIS defines a minimum area of production (20 hectares) to guarantee a sustainable provision of pastures over seasonal- and 2–3 year droughts. Farmers set aside an area for Livestock-Forest-Pasture integration (silvopasture), and intensively cultivate hay and forage, mainly *Opuntia-Ficus Indica* (prickly-pear cactus). Livestock management includes optimal herd sizing to ensure sustainable production in the long run without the depletion of natural resources, especially soil, and a set of best animal management practices. Farmers also organize their farms to include a management center designed to promote sustainable intensification of livestock production and reduce animal heat stress. As needed, they construct wells, water cisterns, and earth damns to ensure family and animal needs during prolonged droughts. Finally, depending on their local conditions, they may purchase recommended small-scale and low-cost machinery, especially tools with a high aggregated labor value, to reduce manual work–this includes technologies like mechanical feed crunchers to process *Opuntia*. All MAIS farmers received technical assistance and training over months in proper implementation and management of the production system.

### 2.2 The benefits of climate resilience interventions

A growing number of studies have evaluated the impacts of agricultural interventions on indicators of agricultural welfare, which includes increases in production, farm income, profits, and decreases in the production costs [21]. In general, access to technical training and the adoption of basic technologies have shown to have a positive impact on agricultural production and farm income. In China, for example, the assistance provided by agrarian scientists in rural communities generated significant benefits on agricultural outcomes [22]. The assistance enabled the diffusion of adequate management practices and overcoming multifaceted yield-limiting factors involving agronomic, infrastructural, and socioeconomic conditions. In the semiarid of Ghana, the combination of credit supply and access to irrigation effectively reduced poverty and the risks associated with climate vulnerability in drought years [23]. In the Brazilian *Sertão*, the access to water for irrigation is scarce, most wells are saline or brackish water, but the adoption of basic types of machinery and fertilizers have shown to have remarkable impacts on family farming production in the region [6].

The adoption of sustainable agricultural practices also benefits the environment and, indirectly, agricultural production. For example, silvopastoral systems in the Caatinga have been able to minimize soil degradation processes, reduce water erosion and losses of nutrients and carbon [24]. In the middle and long-term, improved environmental conditions may increase yield and farm income. For example, Araujo et al. [18] show how increasing the percentage of natural lands in the Caatinga may increase biomass energy production, maintain the flow of essential ecosystem services (such as groundwater stocks), and improve food production.

The benefits of a climate resilience program like the MAIS may also go beyond those captured by traditional indicators of agricultural production and income. For example, the access to information about soil and water conservation, local market opportunities, livelihood diversification, and adaptive household capacity can improve social capital and have spillover effects on life conditions [25]. Strategies that increase agricultural labor productivity may also reduce the farmers’ exposure to high temperatures, reducing heat stress, and improving working conditions [26]. For those farmers who rely on subsistence agriculture, the adoption of adaptive strategies may also be a key factor to increase land productivity and guarantee food security locally [27, 28].

Although household income has been widely used to evaluate the impacts of public policies on quality of life, measures of SWB have attracted growing interest in the literature. Wellbeing encompasses multi-dimensional aspects and gives a sense of how people’s lives are evolving. SWB is self-reported measures of the individuals’ perception about their living conditions that simultaneously incorporate subjective and objective perceptions of life, such as health, comfort, and wealth [29]. One main advantage of measures of SWB in the evaluation of social programs is that they assess more general aspects of the social life, such as life satisfaction and worries with the past and future life conditions [30]. Measures of SWB have been shown to be an effective way to evaluate the perceived benefits of policies targeted to the poorest in developing countries [31]. Subjective measures have also been successfully employed to assess food security situations among impoverished family farmers [5]. Because family farmers in the *Sertão* are subjected to unpredictable production conditions and environmental risks that are not easily captured by traditional socioeconomic indicators, we expect that SWB measures may better capture farmers’ perceptions with improvements in their income and food security, quality of work, and life in general.

The indicators of SWB can be used for the evaluation of policies in many domains, but they are not without caveats. One main concern is that the participation in the program may alter preferences, perceptions, and expectations, which are related to the subjective evaluations of wellbeing (Hawthorne effect) [32]. In this respect, SWB evaluations should also be validated by comparing the convergence of responses with other indicators related to the same concept [33]. For example, changes in the subjective evaluations of income satisfaction and working conditions may be related to improvements in the adoption of new production practices, such as hiring labor and labor-saving technologies.

## 3. Material and methods

### 3.1. Sample design

The survey used in this study involved no risk of physical, informational, or psychological harm to individuals who participated in the interviews. Data were stripped of identifying information, and research subjects did not include vulnerable or dependent groups. Respondents voluntarily agreed (verbal consent) to participate in the survey and answer questions about their agricultural practices, with an option not to respond available for all questions. We did not ask for ethical consent because the institutional review board in Brazil did not require ethnical consent for surveys in applied social sciences at the time that this survey was developed.

Between 2016 and 2018, the MAIS program assisted 100 family farmers in their milk and sheepmeat production. The non-profit organization responsible for the MAIS program (Adapta Sertão) conducted a survey in the Jacuípe Basin in 2015 (henceforth, survey 2015), one year before the implementation of the MAIS program. The aim was to understand the production practices in the region and better select the 100 farmers to receive the MAIS program. The selection of the MAIS farmers was partially random. Fifty farmers were strategically (non-randomly) selected among those with the best-perceived likelihood for success. Based on information collected in the survey 2015, the Adapta Sertão ranked the farmers using a score (henceforth, Adapta score) containing seven main dimensions, each one ranging from 0 to 10: education; family structure; technical training; financial resources; market integration; access to water; land area and management (described in Table 2). The selection of the other 50 farmers was based on: i) a random selection of those farmers who met threshold criteria determined by Adapta Sertao, but who were not among the Adapta selection; ii) farmers recommended by the local cooperative and rural associations.

We conducted a follow-up survey among the MAIS and non-MAIS farmers between October 2017 and January 2018 (henceforth, survey 2018), a few months before the *Adapta Sertão* ended its technical intervention. Participation in the survey was voluntary and did not involve any risk of informational harm to individuals. The initial idea was to follow 100 adopters (treatment group) and a group of 100 non-adopters (control group) before (survey 2015) and after the technical intervention (survey 2018). A secondary goal of the project at its outset was to assess the targeting ability of the organization by comparing the 50 randomly-selected farmers with the 50 Adapta-selected farmers (and comparing both to control farmers). But most farmers selected to participate in the MAIS withdrew the program even before receiving the treatment (non-compliers) because, in the wake of the national financial crisis, the Brazilian Government failed to provide subsidized credit to finance the activities (technical assistance and loans for purchases and on-farm improvements). New farmers were then selected to participate in the MAIS, and now the selection was mainly based on the recommendation of other farmers and local leaderships. These new MAIS farmers were not interviewed in the survey 2015, compromising our study design.

To address these issues as well as possible, we surveyed 201 farmers in 2018 (survey 2018): 94 MAIS and 107 non-MAIS farmers. We pre-selected the non-MAIS farmers to minimize the selection bias caused by the non-random designation of the treatment. We used the data provided by the survey 2015 to fit a logistic regression for the probability of being selected by the MAIS system. Our dependent variable was the log of odds of participation in the MAIS system, where the participation was measured by a binary variable for treatment (MAIS). The independent variables were the Adapta scores and a binary variable that assumes 1 when the farmer was a member of the local cooperative. Next, we predicted the probability of participation in the MAIS program for all farmers. The survey 2018 prioritized the selection of those non-MAIS farmers with the highest probabilities, or those non-MAIS farmers most similar to the MAIS farmers along with the program selection criteria. We provided a list containing 130 non-MAIS farmers to be interviewed, but only 87 were found in the field survey. We then randomly selected the other 20 non-MAIS farmers living in nearby localities.

Here we present an analysis based on two non-mutually exclusive data sets derived from the 2015 and 2018 surveys. The first is the cross-sectional sample described above with 94 MAIS farmers and 107 non-MAIS farmers (survey 2018). The second is a panel dataset that includes the 26 MAIS farmers (*compliers*) and 87 non-MAIS farmers included in both waves of survey data collection (henceforth, panel 2015–2018). The farmers in the panel 2015–2018 are those followed up in the survey 2015 (*t* = 0), before the program implementation, and survey 2018 (*t* = 1). Although panel data have obvious value, our analysis here places more emphasis on the survey 2018, since the number of MAIS farmers in the panel 2015–2018 is small.

### 3.2. Outcome variables

We examine the impact of the MAIS on three sets of outcomes that range from very proximate to the intervention (are farmers using the technologies, as intended?) to further downstream (has the MAIS translated into improved welfare?) (Table 1). These outcome categories are (i) production practices; (ii) land management; (iii) income and subjective wellbeing.

**Table 1:** Average values of outcome variables (standard deviation between parentheses).

Variable		Definition	Survey 2018			Panel 2015–2018		
			MAIS	non-MAIS	* *	MAIS	non-MAIS	* *
*Production Practices*								
	Hired labor	1 if farm hired labor, 0 otherwise	0.660	0.393	***	0.538	0.489	
			(0.476)	(0.491)		(0.503)	(0.501)	
	Brush cutters or shredders	1 if farm has brush cutter or shredder, 0 otherwise	0.851	0.617	***	0.731	0.598	+
			(0.358)	(0.488)		(0.448)	(0.492)	
	Hay storage	1 if farm has structure of hay storage, 0 otherwise	0.638	0.421	**	0.346	0.282	
			(0.483)	(0.496)		(0.480)	(0.451)	
	Soil treatment	1 if farm uses fertilizer, manure or soil corrective, 0 otherwise	0.840	0.561	***	0.788	0.718	
			(0.368)	(0.499)		(0.412)	(0.451)	
	Disease control	1 if farm controls animal disease, 0 otherwise	0.245	0.103	**	0.346	0.253	
			(0.432)	(0.305)		(0.480)	(0.436)	
*Land Management*								
	Capoeira	Hectares with capoeira	13.45	5.184	*	3.144	4.067	
			(40.260)	(11.83)		(7.081)	(7.059)	
	Caatinga	Hectares with caatinga	8.412	2.165	**	1.887	1.878	
			(20.700)	(5.710)		(5.332)	(5.402)	
	Forage	Hectares with forage	4.828	0.416	**	0.462	0.791	
			(14.530)	(1.098)		(0.954)	(2.367)	
	Opuntia	Hectares with Opuntia	1.133	0.517	***	2.112	1.324	
			(1.269)	(0.847)		(5.137)	(4.404)	
	Reforestation	Hectares for reforestation	1.178	0.832		0.530	0.950	
			(2.427)	(2.695)		(1.287)	(2.635)	
*Income & Wellbeing*								
	Farm income	Farm income in the last year	34,194	17,555	***	24,697	17,244	+
			(38,213)	(27,867)		(26,300)	(23,987)	
	Income Satisfaction	1 if the income improved in the last 2 years, 0 otherwise	0.723	0.505	**	-	-	
			(0.450)	(0.502)		-	-	
	Food Satisfaction	1 if the quantity food improved in the last 2 years, 0 otherwise	0.468	0.449		-	-	
			(0.502)	(0.500)		-	-	
	Work Satisfaction	1 if the quality work improved in the last 2 years, 0 otherwise	0.766	0.467	***	-	-	
			(0.426)	(0.501)		-	-	
	Life Satisfaction	1 if the quality life improved in the last 2 years, 0 otherwise	0.660	0.495	*	-	-	
			(0.476)	(0.502)		-	-	

Source: Survey data

*** *p* for difference between MAIS and non-MAIS farmers < 0.001

** *p*<0.01

* *p*<0.05

+ *p*<0.10

The first group of indicators focuses on the main production practices among family farmers in the Brazilian semiarid region: hiring farm workers, use of brush cutters and shredders, access to a structure to store hay, soil treatment in pastures and control of diseases in the animals. These management adaptations are all directly encouraged under the MAIS system, so these outcomes represent, to some extent, a technical validity check. The share of MAIS farmers with permanent or temporary workers was 27 percentage points higher than that of non-MAIS farmers in the survey 2018 (62 of 94 MAIS farmers versus 42 of 107 non-MAIS farmers). Both milk and sheep meat production are labor-intensive activities, and the hiring of manual labor is a vital component to increase production. The use of brush cutters or shredders may to some extent replace the use of labor and is also higher among MAIS farmers: 85% of the MAIS farmers (80 farmers) had access to one of these technologies in the survey 2018, versus 62% of non-MAIS farmers (66 farmers). These technologies are widespread in the region, and the adoption was promoted as part of the MAIS system as a cost-effective strategy to reduce manual labor requirements. The use of a structure for hay storage, which is crucial for feeding animals during prolonged periods of drought, was 22 percentage points higher among MAIS versus non-MAIS farmers (60 MAIS versus 45 non-MAIS). We also found that MAIS farmers are far more likely to have adopted soil treatment in pastures and control of diseases in the animals than non-MAIS farmers in the survey 2018: 79 MAIS farmers (84%) used fertilizer, manure or soil corrective (versus 60 non-MAIS farmers, or 56%); and 23 MAIS farmers (24%) controlled diseases in the animals (11 non-MAIS farmers, or 11%) by strategies of deworming, sanitation, vaccination, and medication.

The second group of outcomes encompasses variables related to land management practices targeted as part of the environmental component of the MAIS system. We analyzed the impacts on (i) area of *capoeira*, secondary vegetation in *Sertão* formed mainly by grass and bushes; (ii) area of forage, or planted vegetation used for grazing or cut to feed to livestock; (iii) area of *Opuntia*, a cactus which is the main source of cultivated nutrition to feed the livestock in the MAIS system; (iv) area of *caatinga*, the original vegetation of *Sertão;* and (v) areas of reforestation, promoted by the project to help farmers use agroforestry to meet national requirements for conservation. Across these indicators, MAIS farmers have larger areas of *capoeira* (13.4 versus 5.2 ha of non-MAIS farmers), more area devoted to foraging crops (4.8 versus 0.4 ha), more cultivation of Opuntia (1.1 versus 0.5 ha); more native area of *caatinga* (8.4 versus 2.2 ha); and more area under reforestation (1.2 versus 0.8 ha). In relative terms, MAIS farmers dedicated 8% of their total area for forage (1% for non-MAIS), 2% for *Opuntia* (1% for non-MAIS), 21% for *capoeira* (11% for non-MAIS farmers), 13% for *caatinga* (4% for non-MAIS), and 2% for reforestation (2% for non-MAIS). MAIS farmers get closer than non-MAIS farmers to compliance with Brazilian Forest Code legislation, which requires that 20% of the total area be left in forest or native vegetation: 15% of the total area covered by caatinga or reforestation among MAIS farmers, and only 6% among non-MAIS. For both MAIS and non-MAIS farmers, the largest share of the farm size was covered by pasture (63% among MAIS and 84% among non-MAIS), usually degraded by inadequate management or lack of conservation.

The third group encompasses the variables directly related to the quality of life: farm income and measures of SWB. The annual average farm income of MAIS farmers was R$ 16,639 (nearly USD 4,200) higher than that of non-MAIS farmers (95% higher) in the survey 2018. We also asked farmers to self-report their perceptions about variations in the sufficiency of income, the quantity of food consumed, quality work, and quality of life in general (these variables were only provided in the survey 2018.) MAIS farmers also reported better perceptions of variation in the last two years for the subjective measures of income (22 percentage points higher), quality of work (30 percentage points higher), and quality of life in general (16 percentage points higher). In turn, there was no significant difference concerning the satisfaction with the variation in the quantity of food in the last two years.

### 3.3. The balance of covariates between MAIS and non-MAIS farmers

Table 2 presents basic descriptive statistics for the explanatory variables used in the sample selection. The variables are divided into three main groups of analysis: Adapta scores, variables related to both the participation in the MAIS and farmers’ outcomes (henceforth, vector **x**); location, variable related to farmers’ outcomes but with no direct (causal) relation with participation in MAIS farmers (henceforth, *Z*_1_); cooperativism, a variable related to the participation of MAIS farmers, but which has no direct relation to farmers’ outcomes (henceforth, *Z*_2_).

**Table 2:** Average values of explanatory variables (standard deviation between parentheses).

Variable		Definition	CS 2018			PD 2015–2018		
			MAIS	non-MAIS	* *	MAIS	non-MAIS	* *
Farmers (*n*)			94	107		26	87	
*Adapta Scores*								
	Education	Average score (0 to 10) for farmer’s education and spouse’s education	4.766	4.229	+	4.115	4.204	
			(2.238)	(1.753)		(1.561)	(1.752)	
	Family	Average score (0 to 10) for farmer’s age, spouse’s age and family members’ age	3.976	3.676		3.823	3.918	
			(2.329)	(2.003)		(2.006)	(2.125)	
	Training	Score (0 to 10) for prior technical training	6.702	6.729		6.154	5.690	
			(4.727)	(4.714)		(4.913)	(4.967)	
	Finance	Average score (0 to 10) for access to credit, debit, nonfarm income and prior farm income	6.335	5.790	*	6.332	6.287	
			(1.559)	(1.681)		(1.251)	(1.490)	
	Market	Average score (0 to 10) for milk traded with cooperative and market	3.785	3.506		4.296	4.242	
			(2.052)	(1.889)		(2.332)	(2.350)	
	Water	Average score (0 to 10) for capacity to access and storage water	5.679	6.466	**	5.527	6.304	**
			(1.966)	(2.062)		(1.625)	(1.535)	
	Land	Score (0 to 10) for land size	8.979	8.467	*	8.538	8.609	
			(1.826)	(1.706)		(1.686)	(1.586)	
*Location*								
	Distance	Distance (km) to the nearest urban center	17.074	15.056	*	14.910	14.074	
*Cooperativism*			(0.826)	(6.155)		(5.415)	(6.400)	
	Cooperative	1 if farmer is member of cooperative, 0 otherwise	0.681	0.458	**	0.788	0.523	***
			(0.469)	(0.501)		(0.412)	(0.501)	

Source: Survey data

*** *p* for difference between MAIS and non-MAIS farmers < 0.001

** *p*<0.01

* *p*<0.05

+ *p*<0.10

Education score (3 for primary education, 7 for secondary education, 9 for uncompleted superior education, 10 for completed superior education); Family score (10 for less than 26 years, 8 for 26–30 years, 6 for 31–35 year, 5 for 36–40 year, 2 for 41–45 years, 0 for 46 years or older); Training score (10 for yes, 0 for no); Finance score—access to credit (10 for year, 0 for no), paid debts (10 for yes or no debit, 0 otherwise) with weight 1, prior farm income (10 for R$24000 or more, 8 for R$ 12000–24000, 6 for R$ 7200–12000, 4 for 2400–72000, 2 for R$ 0–2400) with weight 3, non-farm income (10 for R$24000 or more, 8 for R$ 12000–24000, 6 for R$ 7200–12000, 4 for 2400–72000, 2 for R$ 0–2400) with weight 1; Market score–mild sold to the cooperative (10 for 71–100%, 8 for 51–70%, 6 for 31–50%, 4 for 11–30%, 2 for 0–10%), and milk sold to the market (10 for 0–10%, 8 for 11–30%, 6 for 31–50%, 4 for 51–70%%, 2 for 71–100%); Water score–months with water in the dam (10 for 13 months or more; 8 for 9–12 months, 6 for 7–8 months, 4 for 5–6 months, 2 for 3–4 months, 0 for 0–2 months) with weight 3, flow of fresh water in the well (10 for 3000 m^3/^h or more, 8 for 1500–3000 m^3/^h, 6 for 1000–1500 m^3/^h, 4 for 500–1000 m^3/^h, 2 for 0–500 m^3/^h) with weight 3, flow of brackish water in the well (10 for 3000 m^3/^h or more, 8 for 1500–3000 m^3/^h, 6 for 1000–1500 m^3/^h, 4 for 500–1000 m^3/^h, 2 for 0–500 m^3/^h) with weight 3, cisterns of 50000 m^3^ (10 for 2 or more, 5 for 1, 0 for none) with weight 3, cisterns of 16000 m^3^ (10 for 2 or more, 5 for 1, 0 for none) with weight 1; Land score (10 for 32 ha or more of total land size, 8 for 16–32 ha, 6 for 8–16 ha, 4 for 4–8 ha, 2 for 1–4 ha, 0 for less than 1 ha).

The Adapta scores were chosen among those indicators that better identified the most productive family farmers in the region. As a result, MAIS farmers tended to be positively selected in terms of some dimensions. For example, the financial score of MAIS farmers was 10% higher than those of non-MAIS farmers in the survey 2018. This score includes access to credit, debits, nonfarm income, and farm income in the five years prior to the program. MAIS farms also tended to have larger areas in the survey 2018 (land score of MAIS farmers 6% higher than that of non-MAIS farmers). The project prioritized farmers with an area larger than 20 hectares to guarantee a minimum sustainable agricultural production.

In turn, we identified a negative relation between the water score and the participation in the program. This score was defined by a weighted average of variables related to the current structure of water storage: months in the year with water in the dam; the flow of freshwater and brackish in the well; the number of small and large cisterns. Access to water during prolonged periods of droughts is the essential resource for agriculture in Sertão. The water score of MAIS farmers was 12% lower than that of non-MAIS farmers in the survey 2018: the average water score was equal to 5.8 for MAIS farmers and 6.5 for non-MAIS farmers. Other Adapta scores (education, family structure, training, and access to market) did not statistically differ between MAIS and non-MAIS farmers. These results suggest that the Adpata Sertão did not target as effectively as may be expected. Factors that were not initially controlled by the organization may have also influenced the selection of MAIS farmers.

The most striking difference between MAIS and non-MAIS farmers was related to the membership in a local cooperative: 66% of the MAIS farmers were members of a local farmer cooperative in the survey 2018, versus 46% of the non-MAIS farmers. To account for this, we created a binary variable to help explain unobservable factors related to the participation in the program. Once we control for the Adapta scores, this variable is not expected to have direct impacts (causal relation) on agricultural production. The region does not have a tradition with cooperativism and rural associativism [34]. The main action of the local cooperative is to facilitate the commercialization of agricultural products into the market, which we already account for through controlling for market access (market score).

Finally, we used the distance to the nearest urban center as a proxy for access to urban commercialization channels. The access to paved roads in Sertão is scarce, and long distances to commercialization channels may largely increase the costs of production. The average distance between the farms and urban centers was 17 km for MAIS farmers and 15 km for non-MAIS farmers. Despite this small difference between MAIS and non-MAIS farmers, this variable did not play any major role in the selection of the program.

### 3.4. Identification strategies

#### 3.4.1. Difference-in-Difference estimator

We initially used the panel 2015–2018 and DID estimators to evaluate the impact of the MAIS on the outcome variables. The units of analysis (*i*) were the 26 MAIS farmers and 87 non-MAIS farmers interviewed in both survey rounds (*t* = 0 for survey 2015 and *t* = 1 for survey 2018).

We want to estimate *δ*, the average impact of the treatment (*T* = 0 for non-MAIS and 1 for MAIS farmers) on the outcome *Y*, controlling for farmers’ unobserved heterogeneity *c*_*i*_:

$$Y_{it}=\alpha +\delta T_{i}+{x\prime }_{it}\beta +\theta_{1}Z_{1_{it}}+c_{i}+dt+\varepsilon_{it}$$ (1)

Where *α* is the intercept; **x** is a vector of control variables that are jointly related to *Y* and *T* (the Adapta scores), and **β** its respective vector of coefficients; *Z*_1_ is an exogenous determinant of *Y* (distance to the nearest urban center), and *θ*_1_ its respective coefficient; *d* is the coefficient for the time period dummy; and *ε* is the idiosyncratic error. Controlling for *Z*_1_ allows us to obtain unbiased estimates for *δ*, even if distance and the designation of the treatment are weakly correlated.

The first-differenced equation gives the DID estimator of *δ*:

$${\Delta Y}_{it}=d+\delta T_{i}+{\Delta x\prime }_{it}\beta +\theta_{1}{\Delta Z}_{1_{it}}+{\Delta \varepsilon }_{it}$$ (2)

Where Δ*Y*_*it*_ = *Y*_*i*1_−*Y*_*i*0_, Δ**x**_*it*_ = **x**_*i*1_−**x**_*i*0_, Δ*Z*_*it*_ = *Z*_*i*1_−*Z*_*i*0_, and Δ*ε*_*it*_ = *ε*_*i*1_−*ε*_*i*0_.

The DID estimator controls the farmers’ unobservable characteristics *c*_*i*_ that may affect both *Y* and the selection in the treatment, which are considered constant over time (agricultural skills, for example). The main limitation of the DID estimator in our study is related to efficiency because the precision of our DID estimates is limited by the small sample of MAIS farmers in the panel 2015–2018. The consistency of the DID estimator also relies on the assumption of parallel trends, or that the evolution over time of the MAIS and non-MAIS farmers would be the same in the absence of intervention. Nonetheless, MAIS and non-MAIS farmers may differ in ways that affect their trends over time, as well as their compositions may change over time.

#### 3.4.2. Propensity score matching

We applied PS methods in our survey 2018 using the 201 family farmers of the survey 2018: 94 MAIS and 107 non-MAIS. The PS minimizes the selection bias in the designation of the treatment [35]. Techniques based on the PS have been widely used to evaluate the impacts of policies on a treatment group [36–38]. The method uses the propensity score *p* to balance the treatment and control groups based on a set of observable characteristics that are related to the outcome and the designation in the treatment [39].

We estimated the average treatment effect on the treated (ATT) using three PS methods [40]: (i) *Nearest Neighbor* (NN): for each MAIS farmer, *five* non-MAIS farmers were selected with the nearest values of *p*; (ii) *Kernel*: each MAIS farmer was matched to several non-MAIS farmers, using weights that are inversely proportional to the distance between the values of *p*; iii) *Inverse Probability Weighting Regression Adjustment* (IPWRA): a two-stage estimation strategy, with a selection model for the participation in the program, *p*, in the first stage, and a model for the outcome variable, weighted for *p*, in the second stage.

The former two methods (NN and Kernel) estimate the ATT by differencing the average value of *Y* for treatment and control groups conditioned on *p*, i.e., the difference between the average *Y* of matched MAIS and non-MAIS farmers. The selection models used in matching methods must include all variables related to the outcome, whether or not they are related to the treatment, to minimize potential bias in the ATT estimates [39]. Our selection models were then defined by a *probit* function given by *p* = *P*(*T* = 1) = *ϕ*(**x**, *Z*_1_, *Z*_2_).

The latter method (IPWRA) estimates the ATT by using weighted regression coefficients, where the weights are the estimated inverse probabilities of treatment. The method obtains consistent estimates even when only one of the two equations (selection or outcome model) is correctly specified, this means, the IPWRA is considered a doubly robust strategy [41]. The variables included in the selection and outcome models do not necessarily need to be the same. We added the explanatory variables **x** and *Z*_1_ in the outcome model, which are directly related to *Y*; and the explanatory variables **x** and *Z*_2_ in the selection model, which are directly related to *T*.

Two main hypotheses must be satisfied to obtain unbiased estimates for the ATT using PS methods: (i) balancing hypothesis; (ii) *conditional independence assumption* (CIA). The first hypothesis assumes that the pre-treatment values of the observable characteristics are independent of the treatment, conditioned on the values of *p* [42]. The CIA assumes that, if the potential results are independent of the participation in the program conditioned on the observable characteristics, then these values are also independent of the *p* [36].

#### 3.4.3. Two-stage estimators

We applied two strategies based on 2S regressions to control endogeneity in our survey 2018: local average treatment effect (LATE) and control function (CF). One main limitation in cross-sectional studies is the lack of control for unobservables that may be endogenous to variable *T* representing the designation in the program (selection bias). In our case, the variable *T* tends to be endogenous because unobservable factors affecting the designation may also affect *Y*, i.e., we tend to have a correlation between the error *ε*_*i*_ and the regressor *T* in the structural equation:

$$Y_{i}=\alpha +\delta T_{i}+{x\prime }_{i}\beta +\theta_{1}Z_{1_{i}}+\varepsilon_{i}$$ (3)

The LATE estimator was obtained through two-stage least squares using an exogenous variable as an instrument for *T* in Eq (3) [43]. The consistency of the LATE estimator relies on three main assumptions. First, the relevance assumption assumes that the instrument has a causal effect on *T*. Second, the exclusion restriction assumes that the instrument affects *Y* only through *T*, i.e., the instrument has no direct impact on *Y* once we control for *T*. Third, the monotonicity assumption assumes that all those who are affected by instrument (positively or negatively) are affected in the same way. In our study, the instrument was represented by *Z*_2_ (membership in the cooperative), which determined the participation in the MAIS (assumption 1) but has no direct impact on the outcome *Y* (assumption 2). The membership in the cooperative may also increase the probability of participation in the MAIS for all farmers (assumption 3). Under these assumptions, the LATE estimates the average causal effect of treatment on an instrument-specific subpopulation. In our case, the LATE estimates the average causal effect of treatment on the subpopulation of MAIS farmers that were members of the cooperative.

In turn, the CF controls the endogeneity of *T* by including a proxy for the correlation between the unobservable factor and *T* in the regression model [44]:

$$Y_{i}=\alpha +\delta T_{i}+{x\prime }_{i}\beta +\theta_{1}Z_{1_{i}}+\rho v_{i}+\varepsilon_{i}$$ (4)

Where *v* is a proxy for the unobservable factors affecting *T* and was obtained from:

$$T_{i}=\pi_{0}+{x\prime }_{i}\varphi +\pi_{1}Z_{1_{i}}+\pi_{2}Z_{2_{i}}+v_{i}$$ (5)

The idea under the CF estimator is that, by including *v* in Eq (4), we obtain an error term *ε* that is uncorrelated with *T*. But we also need an additional exogenous regressor in the selection model (Eq 5), which in our case was the same variable used as an instrument in the LATE estimator: membership in the local cooperative. One main advantage of the CF over the PS methods is the greater robustness to misspecification of the conditioning variables, i.e., our variables **x**, *Z*_1_ and *Z*_2_ [45]. We also checked to what extent endogeneity may be a major concern by testing the significance of the coefficient *ρ* in Eq (4). If *ρ* = 0, then *T* is exogenous, and the ATT can accordingly be estimated by controlling only by the observable variables **x** and *Z*_1_.

#### 3.4.4. Oaxaca-Blinder decomposition

Our final empirical strategy applied the Oaxaca-Blinder (OB) decomposition to our survey 2018 [46, 47]. The OB decomposition estimates one outcome model for each treatment (subscript *T*) and control groups (subscript *C*):

$$Y_{T_{i}}=\alpha_{T}+{w\prime }_{T_{i}}\beta_{T}+\varepsilon_{T_{i}}$$ (6)

$$Y_{C_{i}}=\alpha_{C}+{w\prime }_{C_{i}}\beta_{C}+\varepsilon_{C_{i}}$$ (7)

The vector **w** includes the determinants of *Y*, i.e., our variables **x** and *Z*_1_. To account for selectivity, we weighted the observations of the treatment group by 1/*p* and the observations of the control group by 1/(1−*p*). This strategy gives a higher weight for the treated observations that are more closely related to the control group and gives higher weights for those controlled observations that are more closely related to the treated group. The next step is to decompose the average difference between MAIS and non-MAIS farmers ($\Delta {\bar{Y}}_{T-C}$) into:

$$\Delta {\bar{Y}}_{T-C}=[\left({\hat{\alpha }}_{T}-{\hat{\alpha }}_{C})+{\bar{w}\prime }_{T}({\hat{\beta }}_{T}-{\hat{\beta }}_{C}\right)]+[{\bar{w}\prime }_{T}-{\bar{w}\prime }_{C}]{\hat{\beta }}_{C}$$ (8)

Where the first component is the unexplained effect, which represents differences due to unobservable characteristics (for example, the knowledge acquired by the technical assistance provided by the MAIS program, which was not measured in our survey); and the second component is explained effect, which represents differences between the outcome indicators that are explained by observable characteristics (human capital and technology, for example). The unexplained effect also offers a robust estimate of the ATT, while the explained effect includes the selection bias [48–50].

One main advantage of the OB decomposition is to allow us to estimate the direct and indirect impacts of the MAIS program on the quality of life. The MAIS program may directly impact the quality of life through improvements in agricultural production and farm income. The direct impacts would be related, for example, to improvements in the use of better production practices and land management. The idea of this empirical strategy is to compare the unexplained component using different groups of control variables in vector **w**. For example, we can compare the unexplained component of the income differences between MAIS and non-MAIS farmers without and with the controls for production practices. While the former estimate (without control) would represent the total impact of the MAIS program (direct + indirect impacts), the latter (with control) would represent the direct impact. The difference between these estimates would represent the indirect impact of the MAIS on farm income that is a result of improvements in production practices. One second advantage of the OB decomposition is to validate the SWB evaluations. For example, we can check to what extent differences between the subjective assessments of MAIS and non-MAIS farmers are related to differences in the objective indicators of production practices and land management.

## 4. Results

### 4.1. The impacts of the MAIS program

Table 3 presents the ATT estimates using the panel 2015–2018 (DID estimator) and survey 2018 (PS and 2S estimators). Estimates in S1 Table refer to the selection model used in the PS methods. We also included traditional Ordinary Least Squares (OLS) estimates for Eq (3) using the survey 2018. The idea is to evaluate to what extent the OLS estimates may be biased due to selectivity. The DID estimates are not available for the SWB indicators because these questions were only applied in the survey 2018.

**Table 3:** Estimates for the impacts of the MAIS (standard deviation between parentheses).

Variable	DID	OLS	NN	Kernel	IPWRA	LATE	CF
*Production Practices*		* *	* *				
Hired labor	0.055	0.202**	0.267***	0.235*	0.180**	-0.198	0.565***
	(0.161)	(0.075)	(0.068)	(0.093)	(0.075)	(0.409)	(0.138)
Brush cutters or shredders	0.388**	0.223***	0.234***	0.116	0.216**	1.125*	0.495*
	(0.142)	(0.064)	(0.061)	(0.079)	(0.067)	(0.487)	(0.232)
Hay storage	0.223	0.155*	0.218**	0.117	0.173*	0.661	0.479**
	(0.149)	(0.078)	(0.069)	(0.097)	(0.080)	(0.438)	(0.166)
Soil treatment	0.102	0.273***	0.280***	0.324***	0.285***	0.035	0.491*
	(0.142)	(0.069)	(0.063)	(0.089)	(0.076)	(0.364)	(0.235)
Disease control	0.031	0.080	0.142**	0.001	0.053	0.602+	0.239***
	(0.131)	(0.055)	(0.052)	(0.077)	(0.067)	(0.341)	(0.053)
*Land Management*							
Capoeira	2.300	6.858	8.261*	9.251+	6.674	-49.842	8.158
	(2.312)	(4.838)	(4.077)	(5.059)	(4.628)	(31.269)	(6.236)
Caatinga	-1.295	5.323*	6.247**	4.146+	5.430*	-4.880	6.945+
	(1.693)	(2.380)	(2.085)	(2.442)	(2.195)	(11.966)	(3.629)
Forage	0.098	3.042**	4.412**	4.489**	4.361**	0.077	4.922**
	(0.383)	(1.057)	(1.409)	(1.603)	(1.510)	(7.626)	(1.717)
Opuntia	-0.056	0.572***	0.616***	0.525**	0.593***	1.603+	0.951+
	(1.570)	(0.160)	(0.151)	(0.185)	(0.157)	(0.936)	(0.535)
Reforestation	0.104	0.407	0.346	0.129	0.117	4.535+	3.025
	(0.573)	(0.392)	(0.364)	(0.501)	(0.431)	(2.601)	(1.926)
*Income & Wellbeing*							
Farm income	20,203**	14,926***	16,639***	17,175***	16,776***	16,233	4,316
	(7,145)	(4,389)	(4,680)	(4,824)	(4,392)	(26,483)	(18,201)
Income Satisfaction		0.270***	0.219**	0.278**	0.193*	0.433	0.721***
		(0.074)	(0.068)	(0.089)	(0.075)	(0.374)	(0.072)
Food Satisfaction		0.053	0.019	0.031	-0.002	0.745	0.243
		(0.079)	(0.071)	(0.096)	(0.074)	(0.477)	(0.219)
Work Satisfaction		0.324***	0.299***	0.276**	0.352***	0.281	0.614*
		(0.073)	(0.066)	(0.091)	(0.073)	(0.370)	(0.242)
Life Satisfaction		0.233**	0.164*	0.288**	0.211**	0.140	0.642***
		(0.076)	(0.069)	(0.097)	(0.073)	(0.379)	(0.167)

*** *p*<0.001

** *p*<0.01

* *p*<0.05

+ p<0.10

DID and OLS estimates include control variables for Adapta scores (**x**) and *distance* (*Z*_1_). NN, Kernel, IPWRA, LATE and CF estimates include control variables for Adapta scores (**x**), *distance* (*Z*_1_), and *cooperative* (*Z*_2_).

As a result of the small sample size, most DID estimates were insignificant at 10%. The exceptions are the use of brush cutters or shredders (the MAIS would increase the use by 39%) and farm income (the MAIS would increase the income by R$ 20,203 annually, nearly USD 5,000). This variation in the farm income is a meaningful result because it means a twofold increase in relation to the income of non-MAIS farmers.

The PS methods using the CS 2018 (NN, Kernel, and IPWRA) provided more precise estimates than those obtained by DID and 2S estimators (LATE and CF). As a result, most estimates were significant at 5%, suggesting that the MAIS generated positive impacts on several indicators. The significance of the LATE and CF estimates was largely compromised by the high standard errors, which are nearly five times higher than those obtained by PS. Nonetheless, the magnitude of the LATE and CF estimates tends to consistent with those obtained by the PS methods.

The impacts of MAIS on farm income were significant at 1% for all PS estimates (ATT around R$ 17,000, nearly USD 4,250). The PS estimates also suggest that the MAIS improved the perception of improvements in the income (ATT between 19 and 28 percentage points), quality of working conditions (between 30 and 35 percentage points), and satisfaction with the general quality of life (between 16 and 29 percentage points). Positive and significant estimates were also obtained by the CF method. In other words, MAIS farmers tended to present better perceptions of variation of their wellbeing in the last two years. But the perception of change in the quantity of food they consumed was not affected. This may be related to the fact that the MAIS program focused on the production of milk and lamb, which does not necessarily have a direct impact on the diversity of food or farmers’ diets.

The PS estimates were also positive and significant on most indicators of labor and technology, production practices, and land management. The CF estimates were reasonably larger than those obtained by PS, while the LATE estimates were inconclusive due to the large standard errors. The most meaningful results were obtained for: use of hired worker (PS estimates between 18 and 27 percentage points, and CF estimate equals to 56 percentage points); use of brush cutters or shredders (PS estimates between 12 –insignificant at 10%—and 23 percentage points, and CF estimate equals to 49 percentage points); use of hay storage (PS estimates between 17 and 22 percentage points, and CF estimate equals to 48 percentage points); use of soil treatment (PS estimates between 28 and 32 percentage points, and CF estimates equal to 49 percentage points); area of *caatinga* (PS and CF estimates between 4 and 7 hectares); area of forage (PS and CF estimates between 4 and 5 hectares); area of *Opuntia* (PS and CF estimates between 0.5 and 0.9 hectare). Insignificant impacts were observed for control of disease in animals, areas of capoeira, and reforestation.

The validity of the PS estimates relies on two main assumptions: (i) balancing hypothesis: the ability of the PS to match MAIS and non-MAIS farmers with similar observable characteristics; (ii) CIA: the participation in the program is not strongly influenced by unobservable variables. The statistics used to test the balancing hypothesis (S2 Table) indicate that the average differences between MAIS and non-MAIS farmers are nearly zero after the matching. We used the method of Rosenbaum bounds to test the CIA for the matching strategies of NN and Kernel [51] (S3 Table). Our results suggest that most ATT estimates are robust to the effects of omitted factors, independent of the matching strategy.

### 4.2. Decomposing the impacts on quality of life

**Fig 1:**
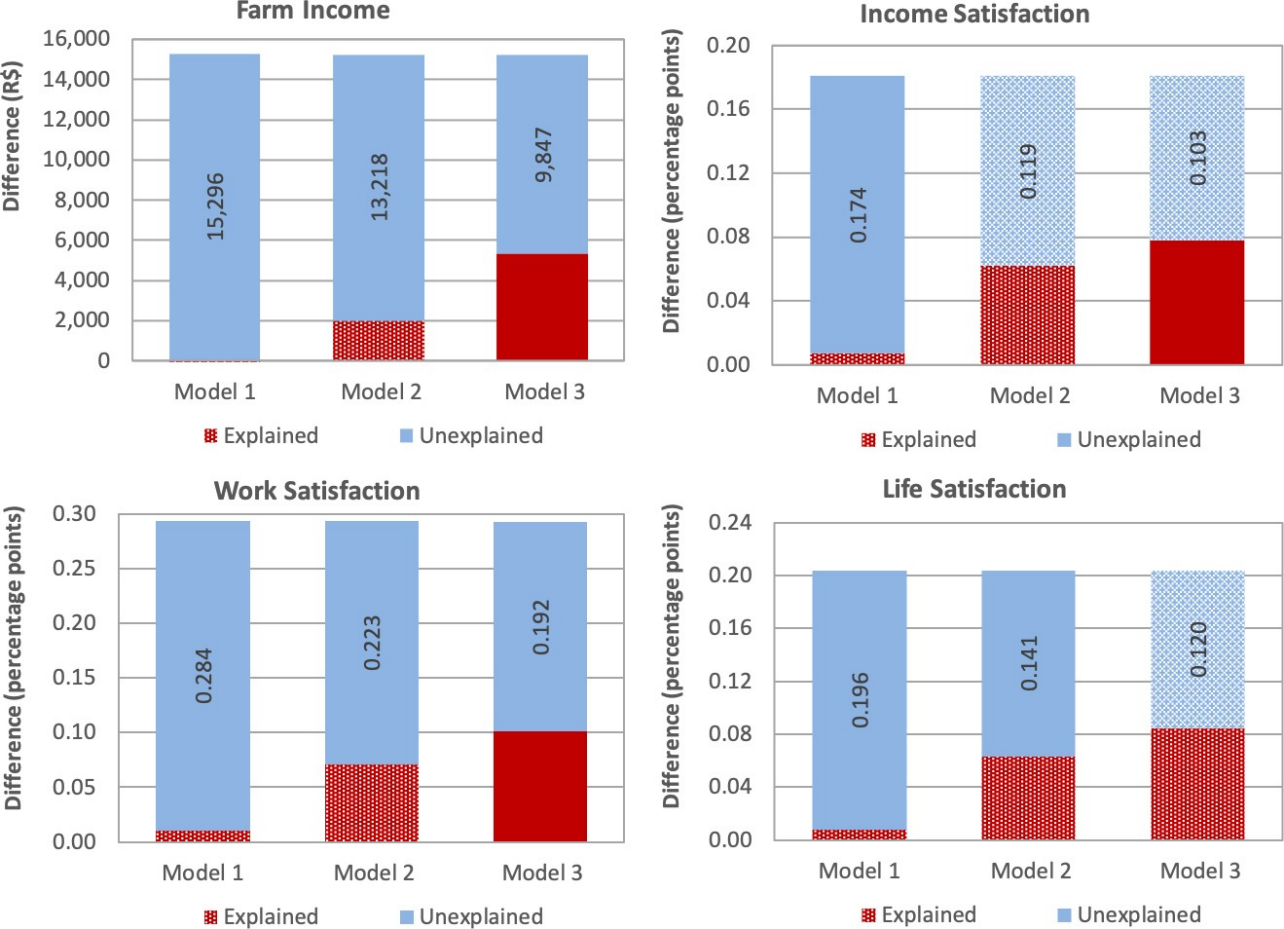
Oaxaca-Blinder decomposition for farm income and subjective wellbeing indicators.

Finally, we decomposed the differences between the indicators of quality of life (income farm and measures of SWB) of MAIS and non-MAIS farmers into (Eq 8): i) explained differences due to observable (control) variables; ii) unexplained differences due to unobservable factors. Fig 1 summarizes the estimates combining different sets of control variables (more information is provided in S4 Table). We only present the estimates for the variables with some component significant at 10%: farm income, variation in the satisfaction with income, work, and life in general. Model 1 controls exclusively by the determinants of *Y* that were not targeted by the MAIS system (**x** and *Z*_1_). The unexplained component in Model 1 can be interpreted as the total impact of the MAIS. For example, the unexplained difference in Model 1 suggests that the total impact of MAIS on farm income was equal to R$ 15,296.

Model 2, which adds controls for production practices proposed by the MAIS, presents an unexplained component of R$ 13,218 for farm income. The difference between the unexplained components of Models 1 and 2 (R$ 15,296−13,218 = 2,078) is a proxy for the indirect benefit of the MAIS program on farm income through improvements in the number of hired workers and better access to technology. Model 3 controls for the whole set of control variables and indicates that nearly 1/3 of the total impact of the MAIS program on farm income (or R$ 5,449) could be indirectly explained by changes in the production practices and land management.

Similar results were obtained by the indicators of SWB. The total impact of the MAIS program on the indicators of SWB ranged from 18 (variation in life satisfaction) and 28 (variation in working conditions) percentage points (Model 1). And the indirect impacts of the program through changes in the production practices and land management (Model 3) ranged between 8 (variation in life satisfaction) and 9 (variation in working conditions) percentage points.

## 5. Discussion and conclusion

This paper adds both empirical and analytical contributions to the literature about the impacts of climate-smart strategies on agriculture production in the developing world. Our main empirical contribution is to emphasize the challenges inherent in, and best solutions for, accurately estimating the impacts of the MAIS program using a small sample of farmers that was not merely a random selection of the population, as would be expected in a pure experimental design. The MAIS program illustrates a relevant case of policy in the developing world, where experimental designs can fall victim to severe budget constraints and political mismanagement, or where randomization is unfeasible more generally.

We present estimates for the impacts of the program using different identification strategies and indicators of agricultural production and quality of life. The indicators include both measures of economic welfare, such as income and production practices, and subjective wellbeing, such as life satisfaction. Each strategy has its associated strengths and limitations, and the set of analyses and outcome indicators complement each other and should be viewed as a whole. The methods based on PS provided more precise estimates, although their consistency relies on the strength of observational variables to control the selection bias. The DID estimates control for farmers’ unobservable factors that are constant over time, but their precision was compromised by the low number of treated farmers in the follow-up study. The accuracy of the estimates based on 2S strategies relies on the strength of our instrumental variable, membership in the cooperative. However, the consistency of these estimates may also be compromised by the small sample size.

We did not find remarkable differences between the OLS and the PS estimates, suggesting that the selection bias in the observable characteristics may not be a severe threat in the impact evaluation. One hypothesis is that the pre-selection of the control group largely attenuated the observable differences between MAIS and non-MAIS farmers. The local cooperative played a major role in the selection of MAIS farmers is likely the main source of bias on unobservables. The DID, LATE, and CF strategies controlled for the selection on unobservables and reinforced many of the positive impacts of the MAIS program, although the precision of these estimates was compromised by the small sample size.

Our main substantive, policy-oriented contribution lies in the finding that basic and low-cost adaptive strategies may have remarkable impacts on income and quality of life of smallholder farmers. Some main consistent achievements were increasing the farm income and access to essential agricultural technologies. This is the first study to evaluate the impacts of a climate resilience program in the Brazilian semiarid region, where family farmers have historically suffered from recurrent and prolonged droughts that have worsened in the last decades. The study provides evidence that fairly simple farm management strategies may be an effective tool for building resilience into rural agricultural systems. MAIS farmers fare better than non-MAIS farmers across several indicators of agricultural production and income, as they also reported better improvements in their work and life conditions. One caveat of the MAIS program is the null impact on the perceptions of improvements in food security. This may be because the program prioritized cash crop productions (milk and sheep meat) rather than the food sufficiency of impoverished farmers–more research into the pathways of impact for cash versus non-cash agriculture for household food security.

We were not able to evaluate the middle- and long-term environmental benefits of the MAIS program. The MAIS also stimulated the reforestation of the native vegetation and the adoption of agroforestry systems, which tend to minimize soil degradation, water erosion, and losses of nutrients and carbon storage in the soil. More sustainable agricultural practices are an urgent need in the *Sertão*, where degraded pastures have extensively replaced the native caatinga vegetation.

A general conclusion is that, albeit still limited in the region, institutional policies aimed to promote access to basic technical guidance and measures to change production practices should be prioritized. Nearly one-third of the impacts of the MAIS on farm income and SWB indicators were related to differences in the use of hired labor, technology, production practices, and land management. For example, one primary strategy disseminated in the program was how to accordingly cultivate densely spaced *Opuntia* cactus. This low-cost strategy focuses on growing forage crops with much higher yields than the traditional cultivation, allowing farmers to feed the animals during periods of prolonged droughts, and avoid overgrazing, land degradation, and land-use change. The other two-thirds of the impact of the MAIS were related to differences that were not directly measured, for example, the technical knowledge disseminated by the program. The MAIS trained extension personnel that was responsible for visiting and advising farmers on a regular and individualized basis, helping farmers to solve several production constraints. This highlights the potentially large role for agricultural extension services in adapting agricultural production systems to changing conditions.

## Supporting information

S1 Table Probit estimates for the likelihood of participation in the MAIS (standard errors between parentheses). (DOCX)

S2 Table Statistics of matching quality (standard errors between parentheses). (DOCX)

S3 Table Rosenbaum bounds sensitivity analysis. (DOCX)

S4 Table Explained and unexplained differences (standard error between parentheses). (DOCX)
