# Supplementary material for: Improving production and quality of life for smallholder farmers through a climate resilience program: An experience in the Brazilian Sertão
Source: PLoS One. 2021 May 21;16(5):e0251531. doi: 10.1371/journal.pone.0251531 (PMC8139507; doi:10.1371/journal.pone.0251531)
Supplement: S1 Table — (DOCX) [file pone.0251531.s001.docx]

**S1 Table** - Probit estimates for the likelihood of participation in the MAIS (Standard errors between parentheses)

| Variable | Estimate |
| --- | --- |
| Education | 0.063 |
|  | (0.052) |
| Family | 0.046 |
|  | (0.046) |
| Training | -0.005 |
|  | (0.022) |
| Finance | 0.130* |
|  | (0.061) |
| Market | 0.041 |
|  | (0.049) |
| Water | -0.145** |
|  | (0.051) |
| Land | 0.135* |
|  | (0.062) |
| Distance | 0.046*** |
|  | (0.013) |
| Cooperative | 0.562** |
|  | (0.205) |
| Observations | 201 |
| Wald χ^2^ (10) | 36.56*** |
| Pseudo R^2^ | 0.141 |
| Correctly classified | 0.632 |
| Sensitivity | 0.575 |
| Specificity | 0.682 |

*** p<0.001, ** p<0.01, * p<0.05, + p<0.1
