# Supplementary material for: Improving production and quality of life for smallholder farmers through a climate resilience program: An experience in the Brazilian Sertão
Source: PLoS One. 2021 May 21;16(5):e0251531. doi: 10.1371/journal.pone.0251531 (PMC8139507; doi:10.1371/journal.pone.0251531)
Supplement: S2 Table — (DOCX) [file pone.0251531.s002.docx]

**S2 Table** – Statistics of matching quality (Standard errors between parentheses)

| Method | Pseudo R2 | LR χ2 | P > χ2 | Mean Bias | Med Bias |
| --- | --- | --- | --- | --- | --- |
| Non-matched | 0.102 | 28.36 | 0.000 | 25 | 27.8 |
| Nearest Neighbor | 0.004 | 1.01 | 0.998 | 4.8 | 5.2 |
| Kernel (Bi-weight) | 0.014 | 3.54 | 0.896 | 8.4 | 7.5 |

Source: Survey data
