# Supplementary material for: Improving production and quality of life for smallholder farmers through a climate resilience program: An experience in the Brazilian Sertão
Source: PLoS One. 2021 May 21;16(5):e0251531. doi: 10.1371/journal.pone.0251531 (PMC8139507; doi:10.1371/journal.pone.0251531)
Supplement: S3 Table — (DOCX) [file pone.0251531.s003.docx]

**S3 Table** - Rosenbaum bounds sensitivity analysis

|  | NN | | Kernel | |
| --- | --- | --- | --- | --- |
| Variable | г | P>\|t\| | г | P>\|t\| |
| *Labor & Technology* | |  |  |  |
| Labor | 2.15 | 0.090 | 2.25 | 0.093 |
|  | 2.20 | 0.102 | 2.30 | 0.106 |
| Brush cutters or shredders | 1.90 | 0.089 | 1.90 | 0.093 |
|  | 1.95 | 0.102 | 1.95 | 0.106 |
| Hay storage | 1.30 | 0.084 | 1.40 | 0.091 |
|  | 1.35 | 0.102 | 1.45 | 0.112 |
| *Production Practices* | |  |  |  |
| Soil treatment | 2.60 | 0.097 | 2.45 | 0.092 |
|  | 2.65 | 0.107 | 2.50 | 0.102 |
| Disease control | 1.30 | 0.094 | 1.35 | 0.088 |
|  | 1.35 | 0.111 | 1.40 | 0.104 |
| *Land Management* | |  |  |  |
| Capoeira | 1.00 | 0.814 | 1.00 | 0.827 |
|  | 1.05 | 0.863 | 1.05 | 0.874 |
| Caatinga | 1.00 | 0.146 | 1.00 | 0.128 |
|  | 1.05 | 0.196 | 1.05 | 0.174 |
| Forage | 1.00 | 0.646 | 1.00 | 0.793 |
|  | 1.05 | 0.717 | 1.05 | 0.846 |
| Opuntia | 1.75 | 0.085 | 1.60 | 0.094 |
|  | 1.80 | 0.103 | 1.65 | 0.115 |
| Reforestation | 1.05 | 0.893 | 1.00 | 0.985 |
|  | 1.10 | 0.923 | 1.05 | 0.991 |
|  |  |  |  |  |
| *Income & Wellbeing* | |  |  |  |
| Farm income | 1.95 | 0.097 | 1.85 | 0.089 |
|  | 2.00 | 0.111 | 1.90 | 0.106 |
| Income Satisfaction | 1.95 | 0.097 | 1.80 | 0.098 |
|  | 2.00 | 0.111 | 1.85 | 0.114 |
| Food Satisfaction | 1.00 | 0.328 | 1.00 | 0.369 |
|  | 1.05 | 0.388 | 1.05 | 0.434 |
| Work Satisfaction | 2.50 | 0.097 | 2.45 | 0.094 |
|  | 2.55 | 0.108 | 2.50 | 0.105 |
| Life Satisfaction | 1.50 | 0.091 | 1.35 | 0.081 |
|  | 1.55 | 0.109 | 1.40 | 0.101 |
