# Supplementary material for: Improving production and quality of life for smallholder farmers through a climate resilience program: An experience in the Brazilian Sertão
Source: PLoS One. 2021 May 21;16(5):e0251531. doi: 10.1371/journal.pone.0251531 (PMC8139507; doi:10.1371/journal.pone.0251531)
Supplement: S4 Table — (DOCX) [file pone.0251531.s004.docx]

**S4 Table** – Explained and unexplained differences (standard error between parentheses)

| Variable | Model 1 | | | | Model 2 | | | | Model 3 | | | |
| --- | --- | --- | --- | --- | --- | --- | --- | --- | --- | --- | --- | --- |
|  | Ex plained |  | Unex plained |  | Ex plained |  | Unex plained |  | Ex plained |  | Unex plained |  |
|  |  |  |  |  |  |  |  |  |  |  |  |  |
| *Income & Wellbeing* |  |  |  |  |  |  |  |  |  |  |  |  |
| Farm income | -86 |  | 15,296 | *** | 1,991 |  | 13,218 | * | 5,361 | + | 9,847 | + |
|  | (1,319) |  | (4,425) |  | (2,340) |  | (5,338) |  | (3,214) |  | (5,267) |  |
| Income Satisfaction | 0.007 |  | 0.174 | * | 0.062 |  | 0.119 |  | 0.078 | + | 0.103 |  |
|  | (0.022) |  | (0.075) |  | (0.039) |  | (0.077) |  | (0.047) |  | (0.079) |  |
| Food Satisfaction | 0.000 |  | 0.041 |  | 0.037 |  | 0.079 |  | 0.009 |  | 0.032 |  |
|  | (0.020) |  | (0.076) |  | (0.039) |  | (0.079) |  | (0.049) |  | (0.077) |  |
| Work Satisfaction | 0.010 |  | 0.284 | *** | 0.071 |  | 0.223 | ** | 0.101 | + | 0.192 | ** |
|  | (0.019) |  | (0.076) |  | (0.050) |  | (0.068) |  | (0.054) |  | (0.073) |  |
| Life Satisfaction | 0.008 |  | 0.196 | * | 0.063 |  | 0.141 | + | 0.084 |  | 0.120 |  |
|  | (0.023) |  | (0.081) |  | (0.048) |  | (0.079) |  | (0.056) |  | (0.078) |  |
|  |  |  |  |  |  |  |  |  |  |  |  |  |
| *Control Variables* |  | | |  |  | | |  |  | | |  |
| Adapta scores | yes | | |  | yes | | |  | yes | | |  |
| Distance | yes | | |  | yes | | |  | yes | | |  |
| Cooperative | yes | | |  | yes | | |  | yes | | |  |
| Production Practices | - | | |  | yes | | |  | yes | | |  |
| Land Management | - | | |  | - | | |  | yes | | |  |

*** p<0.001, ** p<0.01, * p<0.05, + p<0.1; Model 1 includes control variables for Adapta scores (**x**) and *distance* (*Z*_1_); Model 2 includes control variables for Adapta scores (**x**), *distance* (*Z*_1_), and production practices; Model 3 includes control variables for Adapta scores (**x**), *distance* (*Z*_1_), production practices, and land management.
